# Combined Effects of 2 Interleukin 28B Polymorphisms on the Therapeutic Outcome of Hepatitis C Patients With Circulating Cryoglobulins

**DOI:** 10.1097/MD.0000000000001409

**Published:** 2015-09-04

**Authors:** Francesco Bellanti, Gianfranco Lauletta, Rosanna Villani, Maria Rosaria Lipsi, Maria Iole Natalicchio, Domenico Sansonno, Gianluigi Vendemiale, Gaetano Serviddio

**Affiliations:** From the Department of Medical and Surgical Sciences, C.U.R.E. Centre for Liver Diseases Research and Treatment, Institute of Internal Medicine, University of Foggia, Foggia, Italy (FB, RV, GV, GS); Liver Unit, Division of Internal Medicine and Clinical Oncology, Department of Biomedical Sciences and Human Oncology, University of Bari Medical School, Bari, Italy (GL, DS); and Department of Clinical Pathology, II Laboratory, Section of Cytogenetic and Molecular Biology, University Hospital “Ospedali Riuniti”, Foggia, Italy (MRL, MIN).

## Abstract

Chronic hepatitis C is commonly associated with extrahepatic manifestations. Cryoglobulins are observed in 40% to 60% of such patients and their presence seems to modify response to therapy. The new antivirals are greatly improving the sustained virological response (SVR); however, their high cost limits the use, leaving pegylated interferon plus ribavirin (PR) still the standard-of-care therapy worldwide. Since PR therapy is burdened with several side effects, pretreatment predictions of patients who are unlikely to respond to this regimen may avoid ineffective treatment. Variants of the interleukin-28B (IL28B) gene correlate with an SVR to PR, and combined IL28B polymorphisms may improve the prediction of treatment outcome.

The potential role of both rs8099917 and rs12979860 IL28B single nucleotide polymorphisms (SNPs) combined with presence of cryoglobulins in predicting SVR to PR in hepatitis C virus (HCV)-chronically infected patients was analyzed in the present study.

Single and combined IL28B SNPs (rs12979860 and rs8099917) were analyzed in 64 chronic HCV patients treated with PR showing circulating cryoglobulins and compared to 108 noncryoglobulinemic subjects to verify the predictive value on the SVR.

The association of rs12979860CC or rs8099917TT with SVR was confirmed in the noncryoglobulinemic group but not in cryoglobulinemic patients. Moreover, the combined determination of both SNPs improved the prediction of SVR in noncryoglobulinemic patients but not in the cryoglobulinemic subgroup.

We report that both single and combined determination of IL28B rs12979860 and rs8099917 SNPs in chronic HCV patients with circulating cryoglobulins treated with PR may have a reduced predictive value of SVR.

## INTRODUCTION

Hepatitis C virus (HCV) infection represents a major global health problem that requires widespread active interventions for its prevention and control. Even though the exact number of HCV infections worldwide is unknown, a recent report suggests that between 64 and 103 million individuals have chronic HCV infection.^1^ HCV infection is characterized by several extrahepatic manifestations, increasing the risk of mortality and morbidity due to nonliver causes. It has been reported that more than 70% of HCV-positive patients may display an extrahepatic condition with a variable clinical impact.^2^ HCV infection should be considered a systemic diseases with multifaceted pathogenetic implications and clinical features. The most common and well-defined HCV-associated disease is mixed cryoglobulinemia (MC), an immune complex-related vasculitis. MC can also be considered a benign, low grade lymphoproliferative disorder sustained by B-cell clonal expansions, able to evolve into a B-cell non-Hodgkin's lymphoma (B-NHL). Even if the presence of circulating cryoglobulins may be demonstrated in 40% to 60% of HCV-infected patients, only in 10% to 15% of them a clear cryoglobulinemic vasculitis (CV) may occur.^2^ In a 10-year prospective study on 343 HCV-positive patients, half of which with detectable circulating cryoglobulins, it was shown that the progression rate to cirrhosis and extrahepatic complications were not different in cryoglobulinemic and noncryoglobulinemic patients.^3^

In the last years, the advent of new antivirals has been greatly improving the sustained virological response (SVR), defined as undetectable HCV RNA 12 or 24 weeks after therapy conclusion, thus reducing disease progression.^4^ However, pegylated interferon plus ribavirin (PR) will remain the standard of care for many HCV-infected patients worldwide,^5^ at least for those with lower grade of fibrosis. Since PR therapy is burdened with several side effects, pretreatment predictions of patients who are unlikely to respond to this regimen may avoid ineffective treatment. Viral load and genotypes as well as the stage of liver disease strongly predict the response to treatment.^6,7^ Moreover, host genetic differences such as age and race may also influence the response to HCV therapy.^8,9^ A genome-wide association study of patients infected by genotype 1 HCV reported that single nucleotide polymorphisms (SNPs) linked to the cytokine IFNλ3 (also known as interleukin 28B, IL28B) may strongly predict the response to PR therapy.^10–13^ λ-Interferons may inhibit HCV in vitro and may trigger an antiviral cascade through the JAK-STAT pathway.^14^ Two biallelic SNPs rs12979860 (CT) and rs8099917 (TG), located upstream of IL28B gene, have a strong association with both spontaneous and treatment-induced HCV clearance.^15^ Both SNPs are in strong linkage disequilibrium, but the allele frequency of rs8099917 differs between populations worldwide so that its predictive power may vary among different cohorts.^12^ Nevertheless, recent findings demonstrate that, in carriers of rs12979860CT, the determination of additional genotype of rs8099917 SNP significantly improves the prediction of SVR.^16,17^

IL28B rs1297860 polymorphism has been evaluated in relation to different HCV infection statuses in a series of patients by using different outcome and including hepatocellular carcinoma, MC, and NHL. The study confirmed the predictive role of IL28B C allele in spontaneous viral clearance but also a weak relation between IL28B T allele and progression to hepatocellular carcinoma, confirming a carcinogenetic model in which IL28B TT genotype could facilitate cancer development by promoting a persistent viral infection. On the other hand, IL28B CC genotype was more frequently observed in patients with MC than in those with hepatocellular carcinoma or NHL, confirming the hypothesis that viral persistence may represent a risk factor for the development of both liver and hematologic malignancies.^18^

The rs12979860/rs8099917 IL28B polymorphisms have also been analyzed in a large, prospective study of HCV-positive patients with and without MC confirming IL28B genotype as a strong independent predictor of response to interferon-based therapy also in MC patients.^19^ The influence of IL28B rs8099917/rs12979860 on the presence of MC and response to PR treatment was retrospectively examined on 541 patients with chronic hepatitis, 175 of whom with MC. Major genotype TT/CC associated with SVR and, interestingly, was also predictive of MC.^20^

We also contributed to defining the potential role of IL28 in HCV patients affected by CV and treated with PR, demonstrating that TT genotype was more commonly observed in cryoglobulinemic patients and that CC genotype was associated with higher frequency with expanded B cell clonalities in the blood, with kidney involvement and B-NHL. Very interestingly, CC genotype was immunologically characterized by a restricted B cell response and clinically associated with higher risk of renal damage and hematologic malignancies.^21^

On these bases, in the present study we aimed to verify the potential role of both rs8099917 and rs12979860 IL28B SNPs combined with presence of cryoglobulins in predicting SVR in HCV-chronically infected patients treated with PR.

## PATIENTS AND METHODS

### Patients

The study cohort included 172 Caucasian chronic hepatitis C patients attended at the CURE (Centro Universitario per la Ricerca e la Cura delle malattie epatiche of the University of Foggia) or at the Institute of Internal Medicine “Baccelli” of the University of Bari. All patients were treated with PR. Treatment duration ranged from 24 to 48 weeks, depending on the individual treatment response and HCV genotype.

The characteristics of the study cohort are represented in Table 1. In the serum of 64 patients (37.2%), circulating cryoglobulins were detected (cryoglobulinemic group), and patients who did not present with circulating cryoglobulins were grouped as noncryoglobulinemic (n = 108). No patients presented with any symptom related to cryoglobulinemia during the study. Overall, 79 patients (45.9%) achieved SVR, defined as undetectable HCV-RNA levels 24 weeks after completion of the therapy. All other patients were classified as patients with non-SVR. The non-SVR cohort included patients with either null/partial response (N = 55) or relapse (N = 39). Null responders were characterized by little or no decrease in viral load, whereas partial responders experienced at least a 2-log drop in viral load during treatment. Relapse was characterized as HCV-RNA undetectable at the end of treatment, but detectable after treatment completion. In the cryoglobulinemic group, a significant prevalence of females, older, diabetic, and cirrhotic patients was observed which probably explained, almost in part, the response rate to therapy. HCV genotypes 1 and 2 were the most represented in both groups, whereas genotype 4 is less common in our geographic area. The study was performed according to the Declaration of Helsinki. All patients gave informed consent.

**TABLE 1:**
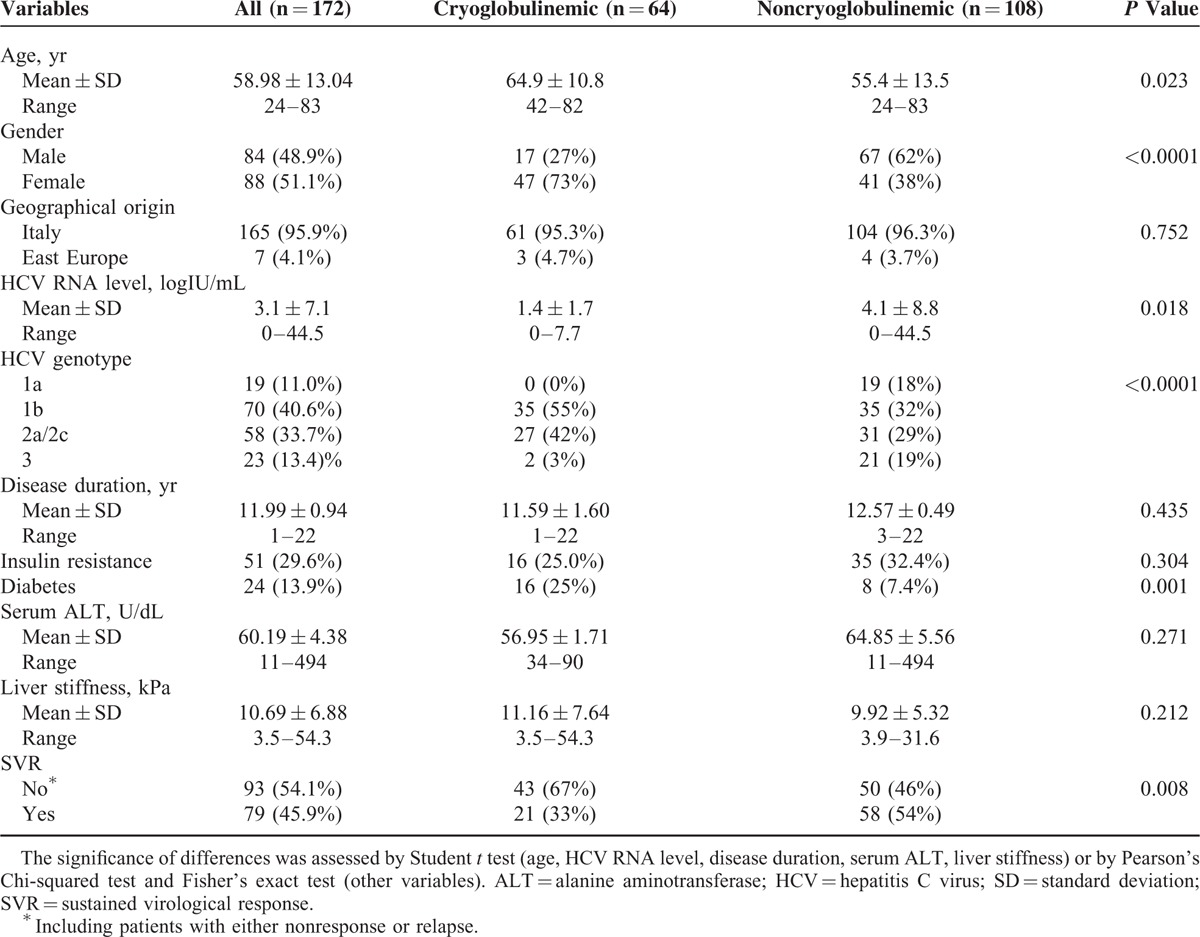
Baseline Characteristics of HCV-Infected Patients According to the Presence (Cryoglobulinemic) or the Absence (Noncryoglobulinemic) of Circulating Cryoglobulins

### Methods

Baseline evaluation included disease history and stage, current signs and symptoms, and previous medications. Physical examination and laboratory values were recorded.

All the DNA samples were genotyped for 2 sets of IL28B SNPs, rs12979860 and rs8099917, specifying by C or T and T or G allele, using a real-time polymerase chain reaction by fluorescent probes (Fast Set IL 28B^©^, Arrow Diagnostics, Genova, Italy), according to the manufacturer's instructions. The assay discriminated the different genotypes: wild-type homozygote (CC, TT), heterozygote (CT, TG), replaced homozygote (TT, GG), for rs12979860 and rs8099917, respectively. Fluorescence data were measured at the end of every cycle by the following profile: denaturation time of 3 min at 95°C, 35 cycle at 95°C for 15 s, and 61°C for 45 s. The results were analyzed using Allelic Discrimination Analysis. The samples were tested by duplicate and the IL28B genotypes were assigned by analysis of the reference cycle numbers for each fluorescence curve.

Serum cryoglobulins were measured, isolated, and purified as described elsewhere.^22^

Liver fibrosis was assessed by transient elastography, performed with the Fibroscan^®^ (Echosens, Paris, France) medical device, using the M or the XL probe after overnight fasting following standard requirements of the Echosense.^23^ A stiffness of 14.8 kPa was considered the cut-off for the diagnosis of liver cirrhosis.^24^

### Statistical Analysis

Data were expressed as count and percentages for qualitative values, and as mean ± standard deviation of the mean (SDM) for quantitative variables. Gaussian distribution of the samples was evaluated by Kolgomorov–Smirnov test. The significance of differences was assessed in contingency tables by Pearson's Chi-squared test and Fisher's exact test. All tests were 2-sided, and *P* values <0.05 were considered to be statistically significant. The odds ratio (OR) and the 95% confidence interval (CI) were calculated. Here, ORs >1 imply a higher chance for SVR relative to the reference category. Multivariate binary logistic regression analysis was used to identify the association of the rs12979860 and rs8099917 variations and haplotypes with SVR. In doing so, adjustments were performed regarding age, gender, HCV-RNA levels, cryoglobulinemia, and fibrosis stage. A further multivariate binary logistic regression analysis was performed to identify the association of the rs12979860 and rs8099917 variations with the presence of cryoglobulinemia, using age, gender, HCV-RNA levels, and fibrosis stage as covariates. The selection of covariates in the multivariate analyses was performed by backward selection, using *P* < 0.1 as a cut-off, with age and sex forced into the model. IL28B SNPs’ comparisons were made using a dominant model, in which patients carrying 1 or 2 copies of minor allele were compared with others. Statistical analysis was performed with the Statistical Package for Social Sciences version 18.0 (SPSS, Inc., Chicago, IL) and the package GraphPad Prism 6.0 for Windows (GraphPad Software, Inc., San Diego, CA).

## RESULTS

### IL28B Genotype Distribution

The overall distribution of IL28B rs12979860 CC, CT, and TT was 36.0%, 50.6%, and 13.4%, and the distribution of IL28B rs8099917 TT, TG, and GG was 58.7%, 33.7%, and 7.6%, respectively. Significant deviations from Hardy–Weinberg equilibrium in genotype distribution were observed for the SNPs as follows: rs12979860: *P* = 3.2 × 10^−5^; rs8099917: *P* = 0.025. However, the Hardy–Weinberg equilibrium in genotype distribution was observed for the IL28B rs8099917 SNP in patients with cryoglobulinemia (*P* = 0.378).

Frequency of genotype distribution of IL28B SNPs was similar between cryoglobulinemic and noncryoglobulinemic patients (Table 2).

**TABLE 2:**
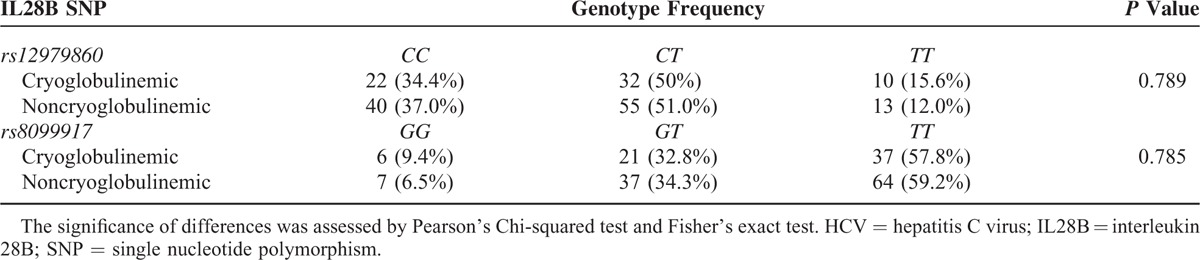
Genotype Distribution of Single IL28B rs12979860 and rs8099917 SNPs (%) in HCV-affected patients, stratified according to the presence (cryoglobulinemic, n = 64) or the absence (noncryoglobulinemic, n = 108) of circulating cryoglobulins

The combined assessment of both SNPs showed frequencies for the most prevalent genotypes, rs12979860CC/rs8099917TT, rs12979860CT/rs8099917TT, and rs12979860CT/rs8099917TG, of 31.4%, 25.0%, and 23.8%, respectively. The remaining genotypes for the combined SNPs were less frequent. When the cohort was stratified according to cryoglobulinemia, no differences were observed in the genotype distribution of combined SNPs (Table 3).

**TABLE 3:**
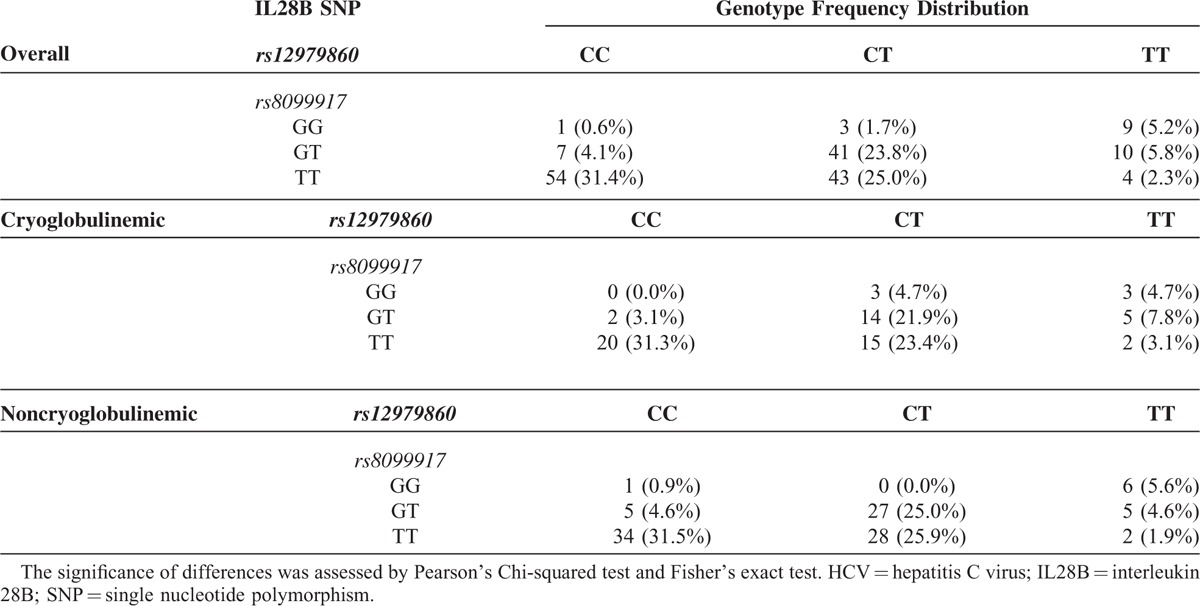
Genotype Distribution of Combined IL28B rs12979860 and rs8099917 SNPs (%) in HCV-Affected Patients, Overall and Stratified According to the Presence (Cryoglobulinemic, n = 64) or the Absence (Noncryoglobulinemic, n = 108) of Circulating Cryoglobulins

### IL28B Genotype and SVR in Chronic Hepatitis C Cryoglobulinemic Patients

Our data show that PR therapy is less effective in cryoglobulinemic HCV patients (SVR was 26.6% vs 73.4% with noncryoglobulins, *P* = 0.008) when all HCV genotypes were considered.

Univariate analysis of factors negatively affecting response to PR in cryoglobulinemic CHC patients revealed that older age (*P* = 0.002), HCV genotype 1, the presence of autoantibodies, cirrhosis and the presence of cryoglobulinemia (*P* = 0.008; OR: 1.637 [1.106–2.422]) were associated with non-SVR, while no association was found for gender, geographical origin, insulin resistance, or diabetes (Table 4). Cryoglobulinemia was most significantly observed in females (OR = 4.518 [2.295–8.895], *P* < 0.0001) and in cirrhotic (OR = 2.053 [1.058–3.981], *P* = 0.04), but was not associated with a specific HCV genotype. A multivariate analysis was performed to verify the most important factors associated with the presence of cryoglobulinemia and showed that female gender was the best independent predictor (OR = 2.540 [1.171–5.189], *P* = 0.018), followed by age (OR = 1.059 [1.027–1.992], *P* = 0.026).

**TABLE 4:**
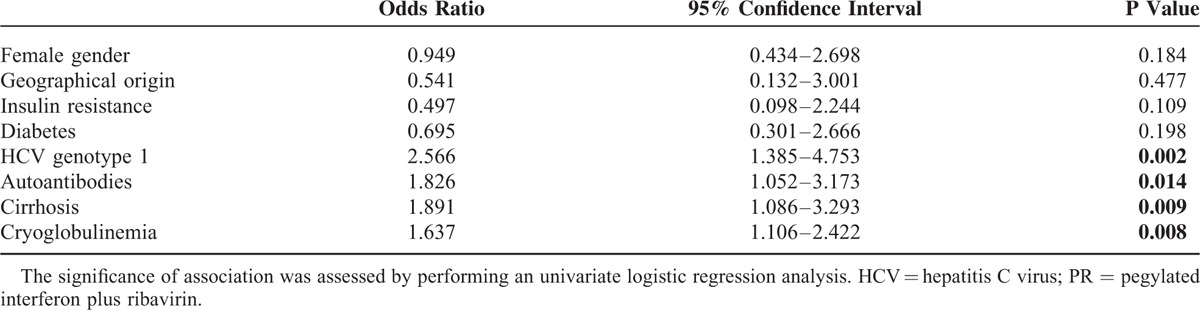
Factors Associated With a Negative Response to PR in Cryoglobulinemic Patients Affected by Chronic Hepatitis C (n = 64)

The association between IL28B SNPs using a dominant genetic model (CC vs CT and TT in rs12979860; TT vs TG and GG in rs8099917) and SVR is reported in Table 5. The rs12979860 non-CC genotypes resulted more frequent in cryoglobulinemic patients, with lower SVR rates. On the other hand, major genotypes CC and TT were associated to SVR in noncryoglobulinemic group.

**TABLE 5:**
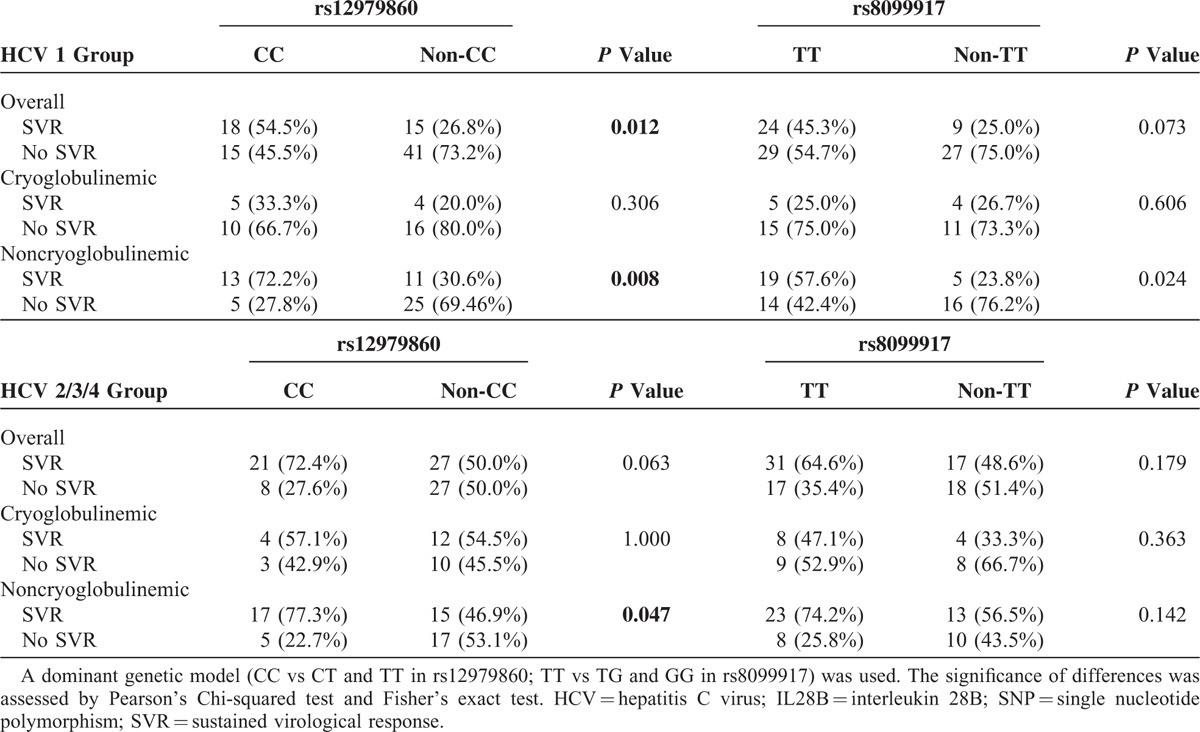
Association Between Single IL28B rs12979860 and rs8099917 SNPs and SVR in Patients Affected by HCV Genotype 1 (Upper Table) or 2/3/4 (Lower Table), Overall and Stratified According to the Presence (Cryoglobulinemic, n = 64) or the Absence (Noncryoglobulinemic, n = 108) of Circulating Cryoglobulins

The association of a single IL28B SNP with SVR after PR therapy revealed a rate of 59.7%, 46.8%, and 26.1% for rs12979860 CC, CT, and TT, and 51.5%, 36.2%, and 46.2% for rs8099917 TT, TG, and GG, respectively. When the overall population was considered in the univariate analysis, the rs12979860 CC was confirmed to be significantly associated with SVR (CC vs CT: *P* = 0.013; CC vs TT: *P* = 0.008) in good agreement with previous reports.^12^

**FIGURE 1:**
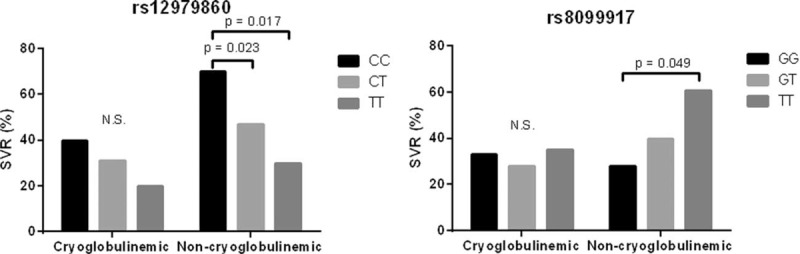
Association between rs12979860 and rs8099917 genotypes and SVR in HCV-infected patients with presence (cryoglobulinemic) or absence (noncryoglobulinemic) of circulating cryoglobulins. Statistical differences were assessed by the univariate logistic regression analysis. HCV = hepatitis C virus; SVR = sustained virological response.

On the other hand, when the overall population was considered, rs8099917TT was not associated with SVR but both rs12979860CC and rs8099917TT confirmed their predictive significance only in noncryoglobulinemic patients (Figure 1).

Tables 4 and 5 also show the association of the best single IL28B SNPs predictor (CC and TT for rs12979860 and rs8099917, respectively) with SVR after PR therapy overall and in cryoglobulinemic and noncryoglobulinemic groups according to the distribution of different genotypes.

Our data confirmed the association between rs12979860 CC polymorphism and SVR in all viral genotypes of noncryoglobulinemic group. Nevertheless, no significant association between IL28 SNPs and SVR was noted in cryoglobulinemic patients even when genotype 1 was individually considered.

The evaluation of rs8099917 SNPs does not seem to increase SVR prediction.

**FIGURE 2:**
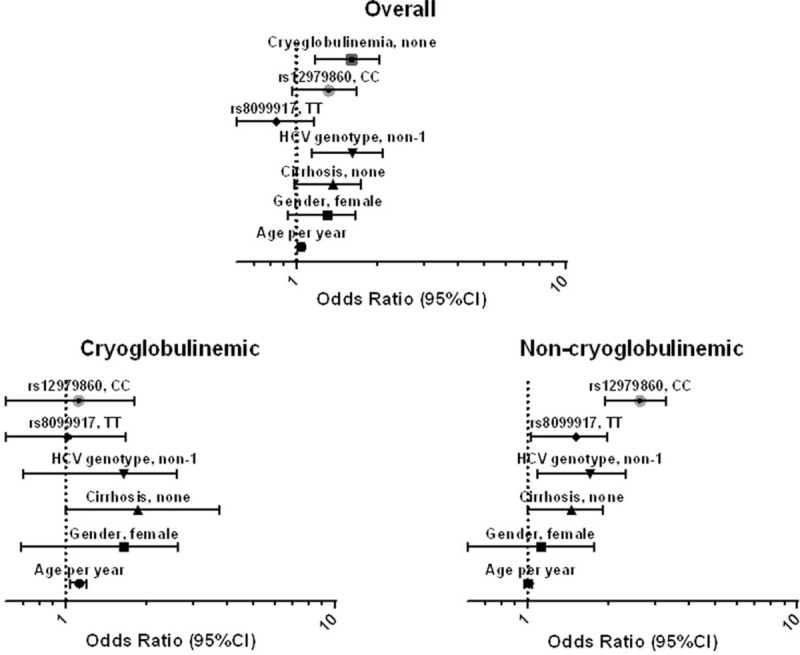
Odds ratios of rs12979860CC and rs8099917TT associated with SVR in a multivariate logistic regression model applied in the entire cohort studied (overall) and in subpopulations of HCV-infected patients with presence (cryoglobulinemic) or absence (noncryoglobulinemic) of circulating cryoglobulins. HCV = hepatitis C virus; SVR = sustained virological response.

A multivariate regression model was applied to verify the best predictors of SVR (Figure 2, upper panel) and showed that HCV genotype is the best predictor of SVR (non-1 vs 1: OR = 1.641 [1.137–2.081]; *P* = 0.013); the presence of serum cryoglobulins was strongly associated with reduced SVR (OR = 1.503 [1.235–2.078]; *P* = 0.008) as well as cirrhosis (OR = 1.27 [1.037–1.781]; *P* = 0.031, cirrhosis vs fibrosis). In the overall population, the multivariate analysis showed a significant association for rs12979860 (CC vs non-CC: OR = 1.328 [0.961–1.669]; *P* = 0.047) but not for rs8099917 (TT vs non-TT: OR = 0.712 [0.610–1.205]; *P* = 0.124).

When the same model was limited to cryoglobulinemic patients (Figure 2, lower panels) our data confirmed that both rs12979860CC and rs8099917TT genotypes maintained the positive prediction only in the noncryoglobulinemic group (CC vs non-CC: OR = 1.330 [2.130–3.380]; *P* = 0.007 and TT vs non-TT: OR = 1.440 [1.070–2.010]; *P* = 0.014 in noncryoglobulinemic; CC vs non-CC: OR = 0.846 [0.610–1.894]; *P* = 0.099 and TT vs non-TT: OR = 0.668 [0.616–1.772]; *P* = 0.096 in cryoglobulinemic).

### Combination of rs12979860 and rs8099917

The combination of the 2 SNPs resulted in 3 major genotypes (rs12979860CC/rs8099917TT, rs12979860CT/rs8099917TT, and rs12979860CT/rs8099917TG) with frequencies more than 20%, which are associated with treatment outcome of at least 20% in the subpopulation of noncryoglobulinemic patients.

Considering the total cohort studied, subgroup analysis revealed that in patients with homozygous rs12979860CC genotype, the additional determination of rs8099917 had no significant effect on the prediction of SVR rate (rs12979860CC/rs8099917TT vs rs12979860CC: OR = 0.983 [0.467–2.067]; *P* = 0.945; rs12979860CC/rs8099917TG vs rs12979860CC: OR = 0.901 [0.185–4.378]; *P* = 0.936). Similarly, in patients with the heterozygous variants of the rs12979860 nonresponder T allele, the pattern of the rs8099917 SNP did not affect the chances of achieving SVR (rs12979860CT/rs8099917TT vs rs12979860CT: OR = 0.614 [0.294–1.281]; *P* = 0.261; rs12979860CT/rs8099917TG vs rs12979860CT: OR = 1.224 [0.569–2.631]; *P* = 0.699).

We also verified if the combination of rs12979860 and rs8099917 might improve the prediction of SVR in the HCV cryoglobulinemic patients where single SNP seemed not predictive. When the subgroup analysis was performed in noncryoglobulinemic patients, the additional determination of rs8099917 to the heterozygous variants of the rs12979860 nonresponder T allele, significantly strengthens the chances of achieving SVR (rs12979860CT/rs8099917TT vs rs12979860CT: OR = 1.317 [1.052–1.834]; *P* = 0.045; rs12979860CT/rs8099917TG vs rs12979860CT: OR = 1.287 [0.945–2.012]; *P* = 0.074) but, once again, no significant association was observed in HCV-positive patients with cryoglobulins (rs12979860CT/rs8099917TT vs rs12979860CT: OR = 1.232 [0.590–2.571]; *P* = 0.707; rs12979860CT/rs8099917TG vs rs12979860CT: OR = 0.817 [0.380–1.757]; *P* = 0.699).

## DISCUSSION

PR has been for a long time the standard of care for chronic HCV infection. HCV infection is also characterized by a peculiar lymphotropism, sustaining an indolent B-cell clonal expansion that represents the pathogenetic substrate of MC.^25^ MC can also be defined as an immune complex-mediated vasculitis potentially determining organ damage and evolving into malignant B-cell NHL. It has been previously observed that the presence of MC may also influence the clinical outcome of HCV-infection,^26,27^ but a successful antiviral therapy based on interferon with or without ribavirin regimens increases the survival independently of MC.^27^

Being HCV the etiologic factor causing MC, the possibility of antiviral therapy with or without B-cell depletion with rituximab is now considered the more rational therapeutic approach.^28^

The use of new antiviral drugs, even though they seem to be highly effective in cryoglobulinemic hepatitis C, is limited; as a consequence, interferon-based therapy still represents the standard therapy in such conditions in many countries. In this context, in order to avoid potential side effects due to interferon,^29^ the possibility to predict the clinical and virologic outcome before starting therapy could be precious in a tailored therapeutic approach.

IL28B SNPs have been indicated as strong predictors of SVR after PR-based therapy,^9–11^ suggesting the possibility of a patient-tailored treatment to limit the risk of toxicity in patients with lower probability of an SVR.

In a series of 1050 HCV-positive patients, IL28B rs1297860C allele was reported as a predictive marker of viral clearance, whereas TT genotype seemed associated with malignant conditions like hepatocellular carcinoma or NHL (even though it was not significant in this latter case).^18^ In addition, it has been previously observed that IL28B genotype is a strong independent predictor of response to IFN-based therapy also in mixed cryoglobulinemic HCV patients.^19^ We have recently reported that rs12979860CC genotype associated with 52.6% of SVR in a cohort of 159 patients with CV, and that CC genotype associated with a restricted B-cell clonal expansion and higher risk of kidney involvement and hematologic malignancies.^21^

In the present study, we investigated the association between the IL28B polymorphic variants in patients affected by chronic hepatitis C and circulating cryoglobulins, and whether combination of IL28B SNPs rs12979860 and rs8099917 might improve SVR prediction in such patients.

It has been clearly demonstrated that the IL28B SNPs are strongly predictive of SVR to PR therapy in patients infected by HCV genotype 1,^10–13^ and accordingly to some authors they may be useful in other genotypes,^30–32^ thus we did not limit the analysis to HCV genotype 1 infection. However, it is worth to note that we registered a very low number of patients infected by HCV genotype 4, which represents a limitation of the present study. Our data reveal similar IL28B genotypes distribution in HCV patients with circulating cryoglobulins as compared to chronic HCV patient without, in good agreement with previous report suggesting that genetic variations in IL28B genes proximity do not influence the development of cryoglobulinemia.^18^ Nevertheless, a different distribution of IL28B genotypes has been reported by a recent study, even though this result may be dependent on the geographical origin of the enrolled patients.^20^

SVR rates resulted lower in HCV positive patients with circulating cryoglobulins. The association between cryoglobulinemia and SVR in chronic hepatitis C has been previously investigated, leading to conflicting results. In fact, association between cryoglobulinemia and higher SVR rates has been reported in a Brazilian cohort^33^; however, 2 recent studies have reported that cryoglobulinemia is negatively associated with SVR in chronic hepatitis C patients.^34,35^ A further study reported that cryoglobulinemia is associated with low SVR rates in symptomatic patients, while asymptomatic MC presents with similar SVR rates than noncryoglobulinemic subjects.^20^ It is conceivable that the discrepancies might be dependent on ethnic differences.^36–40^ Moreover, this study confirms that IL28B SNPs are associated with SVR, irrespective of HCV genotype, in patients with no circulating cryoglobulins but not in those with presence of serum cryoglobulins.

The combined assessment of all SNPs showed frequencies for the most prevalent genotypes rs12979860CC/rs8099917TT, rs12979860CT/rs8099917TT, and rs12979860CT/rs8099917TG that reached the highest values of frequency (23% to 32%) as compared to the variants rs12979860CC/rs8099917GG and rs12979860CT/rs8099917GG, that had a frequency rates <2%. A previous observation found that in patients with a heterozygous variant for the nonresponder rs12979860 T allele, the additional genotyping of rs8099917 significantly improved SVR prediction, suggesting the combined determination of rs12979860 and rs8099917 genotype as a further diagnostic procedure for improving treatment decisions.^16^ Even though only indicative, the present observation seems to support the utility of the additional genotyping of rs8099917 in patients without circulating cryoglobulins. On the contrary, our data suggest that the combined determination of rs12979860 and rs8099917 does not increase the predictive value of the SVR to PR in chronic hepatitis C patients with circulating cryoglobulins.

Boglione et al^20^ found the combined assessment of both IL28B SNPs useful in MC; nevertheless, the Authors included a consistent group of Egyptian patients presenting with different distribution and predictive value of IL28B with respect to Caucasians.

In conclusion, we report that both single and combined determination of IL28B rs12979860 and rs8099917 SNPs in chronic HCV patients with circulating cryoglobulins treated with Peg-IFN plus Ribavirin may have a reduced predictive value of response. This could be dependent on several hypotheses, such as the different genetic background as well as other viral reservoirs like mononuclear cells that could explain a longer viral persistence in peripheral blood mononuclear cells with a compartmentalization of viral quasi-species.^41,42^

Considering the adverse effects of PR-based regimen therapy we hope that, almost in cryoglobulinemic patients and in those affecting by any kind of HCV-dependent lymphoproliferative disorder more choice will likely mean greater options both in terms of different regimens to choose as well as perhaps lower costs. This enormous progress is something that we are hopeful we will all be able to take advantage of very soon.
